# Conservation in the Context of Climate Change: Practical Guidelines for Land Protection at Local Scales

**DOI:** 10.1371/journal.pone.0080874

**Published:** 2013-11-20

**Authors:** Kevin Ruddock, Peter V. August, Christopher Damon, Charles LaBash, Pamela Rubinoff, Donald Robadue

**Affiliations:** 1 The Nature Conservancy, Rhode Island Chapter, Providence, Rhode Island, United States of America; 2 Environmental Data Center, Department of Natural Resources Science, University of Rhode Island, Kingston, Rhode Island, United States of America; 3 Coastal Resources Center, Graduate School of Oceanography, University of Rhode Island, Narragansett, Rhode Island, United States of America; University of Saskatchewan, Canada

## Abstract

Climate change will affect the composition of plant and animal communities in many habitats and geographic settings. This presents a dilemma for conservation programs – will the portfolio of protected lands we now have achieve a goal of conserving biodiversity in the future when the ecological communities occurring within them change? Climate change will significantly alter many plant communities, but the geophysical underpinnings of these landscapes, such as landform, elevation, soil, and geological properties, will largely remain the same. Studies show that extant landscapes with a diversity of geophysical characteristics support diverse plant and animal communities. Therefore, geophysically diverse landscapes will likely support diverse species assemblages in the future, although which species and communities will be present is not altogether clear. Following protocols advanced in studies spanning large regions, we developed a down-scaled, high spatial resolution measure of geophysical complexity based on Ecological Land Units (ELUs) and examined the relationship between plant species richness, ecological community richness, and ELU richness (number of different ELU types). We found that extant landscapes with high ELU richness had a greater variety of ecological community types and high species richness of trees, shrubs, and herbaceous plants. We developed a spatial representation of diverse ELU landscapes to inform local conservation practitioners, such as land trusts, of potential conservation targets that will likely support diverse faunas and floras despite the impact of climate change.

## Introduction

Climate change will alter the composition of the plant and animal communities [1]–[9]. This appears to be happening already as evidenced by studies comparing historic and current range distributions [3]–[8]. The implication is that the ecological communities of a present-day landscape may be quite different in the future when climates are markedly different. For example, depending on which climate change model is used, the climate of the state of Rhode Island USA in 2100 will be similar to the current climate of the mid-Atlantic or southeast United States [10]. This presents a challenge for conservationists working to protect biodiversity: will the current portfolio of conserved lands in a region be effective in protecting biodiversity when the composition of plant and animal communities has profoundly changed [5], [11]? Put another way, where are the important lands to protect now that will have high value in protecting biodiversity when climates and local ecosystems are markedly different in the future?

Hunter et al. [12] argued for the conservation of ecological communities rather than specific species and termed this approach “coarse-filter” conservation. Moreover, they specifically advocated for the protection of those geophysical characteristics (soils, climate, topography) which are the “arenas” of different plant and animal communities. Recently, Anderson and Ferree and others [13]–[15] argue that since species composition of ecological communities will be changing over the decades as a result of climate change and other forcing factors, we should focus on conserving geophysical settings that support diverse, interesting, or important plant and animal assemblages. Using the metaphor of the ecological theater advanced by Hutchinson [16], they call for a coarse filter approach to protecting the stage (geophysical setting), not just the specific actors (species) [13]–[15]. Anderson and Ferree found a strong positive correlation between geophysical setting, as defined by bedrock geology and landform, and plant and animal diversity at a statewide scale in the northeastern United States. Similar relationships between geophysical diversity and biodiversity have been observed at finer scales [17], [18].

The goal of this analysis was to determine if the method used by Anderson and Ferree [13] at state-scales (1,000 s sq km) could be down-scaled to perform in smaller areas with greater spatial resolution in order to provide conservation planning guidance to local conservation organizations, such as land trusts, land conservancies, municipalities, and non-governmental organizations (NGO) who preserve small parcels of land (10 s to 100 s hectares) to protect local biodiversity and ecosystem services. Specifically, we are interested in determining if it is possible to identify landscapes that will support a large number of ecological communities and different plant species (and presumably animal species as well) as climates change. The basis of our analyses are “ecological land units” (ELU, [13], [19], [20]) which are relatively homogeneous areas of a particular landform (e.g., hilltop, slope, and valley) and geomorphological composition. ELUs' are sometimes called “Land Facets” [14], [21]. We evaluate the efficacy of our measure of ELU richness to predict high biodiversity by comparing the local variation in geophysical settings with patterns of plant species diversity in biological refuges owned by the Audubon Society of Rhode Island. We also involved Rhode Island state and municipal officials and local land trusts to explore how to present landscape scale patterns of ELUs in a manner that is usable and interpretable by conservation practitioners who have limited technical resources to draw upon for conservation planning.

## Materials and Methods

Our choices of data and technical procedures to map ELUs were based on the objective to make implementation possible by local conservation organizations who do not have extensive resources in geospatial data processing or staff ecologists. Furthermore, we endeavored to make the resolution of ELUs practical for use at local scales to assist in the identification of priority parcels for land trusts or community conservation organizations [22]. When possible, we used off-the-shelf data that are readily available from reliable sources. All data processing was done using ArcGIS 10 software (Environmental Systems Research Institute, Redlands, CA).

### Sources of Data

Landform was obtained from a photogrammetrically-derived digital terrain model [23] depicting a bare-earth surface downloaded from the Rhode Island Geographic Information System [RIGIS] data repository (http://www.edc.uri.edu/rigis). The raw data were in a TIN (triangulated irregular network) data model stored in an esri terrain file format [24]. These elevation data have a vertical accuracy of approximately 3 m and were based on mass point elevations with a mean spacing of 6.3 m in landscapes with varying topography. The elevation terrain model was converted to a raster digital elevation model with a cell size of 3 m. These data are technically consistent with digital elevation models available for much of the United States (National Elevation Data, NED) from The National Map data collection maintained by the United States Geological Survey [http://nationalmap.gov/, 25].

Soil drainage and surface texture were obtained from SSURGO (state soil survey geographic database) obtained from the United Stated Department of Agriculture Natural Resources Conservation Service [26]. In Rhode Island, SSURGO data are mapped at a scale of 1∶15,840 and have a minimum polygon size of approximately 0.2 ha.

Open water polygons used in the derivation of ELUs were obtained from the RIGIS database. These data were delineated from 1∶5,000 digital orthophotography in 1997 and have a minimum polygon size of 0.1 ha. These data are generally consistent with the surface hydrography (National Hydrographic Data, NHD) available from The National Map data collection maintained by the United States Geological Survey (http://nationalmap.gov/).

**Figure 1:**
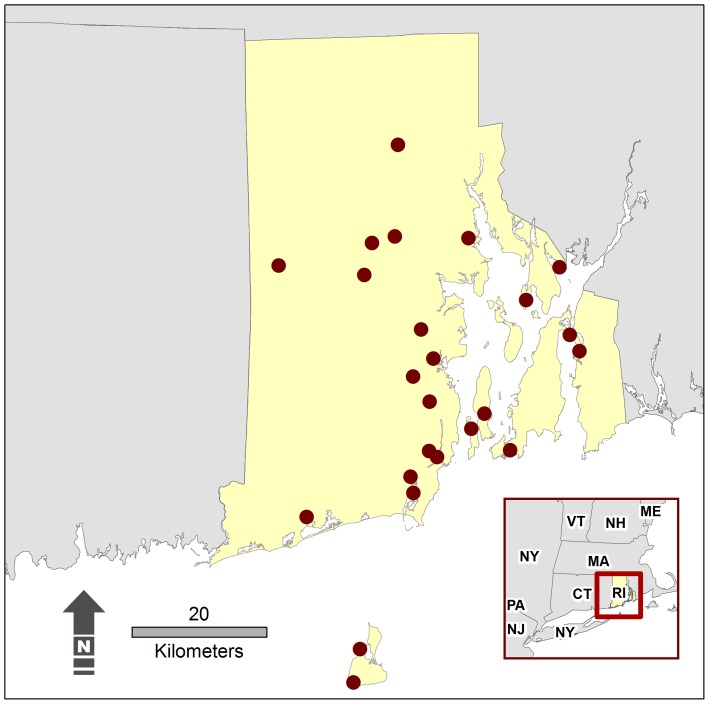
Audubon Society of Rhode Island Refuges. Locations of refuges where vascular plant inventories were conducted.

Biodiversity data were obtained from plant surveys conducted in 1993 and 1994 on Audubon Society of Rhode Island (ASRI) refuges [17], [27]. Comprehensive inventories of all species of vascular plants (herbs, shrubs, trees) and measurement of the number of different plant communities were conducted by two experienced plant taxonomists on 24 refuges ranging in size from 1.4–60 ha (Figure 1). All surveys were based on an individual-based sampling protocol [28] and were performed by the same botanists at a survey rate of 2 ha/hr. Inventories were conducted on each refuge until no new species were encountered when fully traversing small areas or no new species were found in 20 person minutes of searching. The ecologists performing the surveys conducted a validation assessment on one Audubon reserve [29]. The two botanists surveyed the site in 18 person hours. A group of 10 experienced plant ecologists surveyed the same site in 60 person hours. The original 18-hour survey contained 92% of the species recorded in the more intensive 60-hour replicate survey. All Audubon refuge surveys were performed by the same two scientists, thus any biases in their surveys were consistent across all refuges. Original field notes by the ecologists surveying the flora of the Audubon refuges are unavailable to us, thus estimating species richness using rarefaction methods was not possible [28].

### Deriving Ecological Land Units

We followed the procedures described in Anderson and Ferree and Fel and others to map ELUs [13], [19], [30], [31]. Rather than using bedrock geology data to define ELUs we used SSURGO soils data because they were available at a more resolute scale (1∶15,840, minimum mapping unit of 0.2 ha) than bedrock geology (1∶100,000) in Rhode Island. Furthermore, soils are often used when mapping ELUs because of their importance in defining plant communities [17], [18], [21], [32]. Following previously published methods in mapping geomorphological heterogeneity [17], [18] and in consultation with soil scientists and plant ecologists, we used two fundamental soil properties - soil drainage class and soil surface texture - in defining ELUs (Table 1). Soil drainage class distinguishes well-drained (dry) and poorly-drained (wet) soils and are important in defining wetland and upland plant communities [17]–[19]. Surface texture of soils (sandy, gravelly, loamy) are a determinant of many different plant associations [32]. Both parameters are included in the SSURGO attribute database. Although we considered each soil polygon to be homogeneous for its soil type, we know that it is possible to have aberrant inclusions of other soil types that are smaller than the minimum mapping unit of the dataset (0.2 ha for RI SSURGO data).

**Table 1:** Soil drainage, soil texture, and landform classes used to identify ELUs.

Soil Drainage Classes	Soil Texture Classes	Landform Classes
Excessively Drained (1000)	Gravelly Sand (100)	Steep Slope (04)
Well Drained (2000)	Sand (200)	Cliff (05)
Poorly Drained (3000)	Loamy Sand (300)	Flat Summit (11)
Variable (4000)	Fine Sandy Loam (400)	Slope Crest (13)
Water (5000)	Silt Loam (500)	Hilltop (21)
	Muck (600)	Hill, Gentle Slope (22)
	Bedrock (700)	NE-facing Sideslope (23)
	Variable (800)	SW-facing Sideslope (24)
	Water (900)	Flat, Dry (30)
		Flat, Wet (31)
		Valley, Toe Slope (32)
		Flat, Base of Steep Slope (41)
		NE-facing Cove (43)
		SW-facing Cove (44)
		Water (51)

The code values in parentheses are the class codes to identify each condition. ELUs are formed by merging these three GIS layers resulting in unique combinations of landform and soil conditions (see Table 3)

The code values in parentheses are the class codes to identify each condition. ELUs are formed by merging these three GIS layers resulting in unique combinations of landform and soil conditions (see Table 3).

Landform is a complex measure representing unique combinations of elevation, slope, aspect, surface curvature, and upslope catchment area. We used the approach described by Fels and Matson [30] and used by others [13], [15], [19], [31] to model landforms. This method relies on slope and landscape position relative to surrounding elevations to define topographic units (e.g., steep slope, flat hilltop, wet flat). We identified 14 different landform conditions (Table 1, see Supplemental Information S1).

Soil drainage class, soil surface texture classes, and landform were combined to incorporate all these factors into a single raster dataset (15.24 m [50 ft] cell size) where every pixel contained the value for soil drainage, texture, and landform class (Table 2).

**Table 2:** Most common ELU categories accounting for 85% land area of Rhode Island.

ELU Code	Description	Area (Sq Km)	Percent Land Area of Rhode Island % Land area of RI
2432	Well drained fine sandy loam on valley/toe slope	382.3	14.5
2422	Well drained fine sandy loam on gentle slope	249.1	9.5
2430	Well drained fine sandy loam on dry flat	234.8	8.9
2421	Well drained fine sandy loam on flat hilltop	143.2	5.5
2423	Well drained fine sandy loam on upper sideslope/rounded ridge	116.7	4.4
2424	Well drained fine sandy loam on SE facing sideslope	112.1	4.3
3421	Poorly drained fine sandy loam on flat hilltop	107.6	4.1
2530	Well drained silt loam dry flat	106.6	4.1
2532	Well drained silt loam on valley/toeslope	105.2	4.0
3621	Poorly drained muck on flat hilltop	102.5	3.9
3422	Poorly drained fine sandy loam on gentle slope	87.9	3.3
2522	Well drained silt loam on gentle slope	83.6	3.2
2521	Well drained silt loam on flat hilltop	73.0	3.2
1122	Excessively drained gravelly sand on gentle slope	56.5	2.1
1132	Excessively drained gravelly sand on valley/toe slope	56.1	2.1
1130	Excessively drained gravelly sand on dry flat	33.9	1.3
1121	Excessively drained gravelly sand on flat hilltop	31.9	1.2
3521	Poorly drained silt loam on flat hilltop	31.9	1.2
1123	Excessively drained gravelly sand on upper sideslope/rounded ridge	30.4	1.2
1124	Excessively drained gravelly sand on SE facing sideslope	29.6	1.1
1430	Excessively drained fine sandy loam on dry flat	23.7	0.9
3622	Poorly drained silt loam on gentle slope	22.4	0.8

### ELU Metrics

To test our hypothesis that areas that contain a large variety of ELU types (high ELU richness) will contain a large variety of plant communities and high biodiversity, we created a raster surface that contained the number of different ELUs in a 457 m (30 pixel, 1,500 ft) radius. The larger the number of unique ELU classes in the moving window [24], the greater the local richness of ELUs. A pixel that is surrounded by the same ELU type would have a value of 1 and indicate a homogeneous landscape. We chose a moving window of 457 m (30 pixels) because this generally corresponds to the area (∼65 ha) that a Rhode Island land trust or other conservation organization might typically be interested in purchasing. We experimented with alternative sizes of moving windows in increments of 10 pixels from 10 to 50 pixels. Small radii (10 pixels, 152 m, 500 ft) did not capture regional patterns of ELU variety very well whereas large radii (50 pixels, 762 m, 2,500 ft) homogenized regional variation in ELUs. The 30 pixel (457 m) radius performed best at capturing ELU variety.

To simplify mapping of ELU richness into discrete classes that could be used to identify areas of high variation in ELUs, we reclassified the ELU richness raster into areas based on standard deviation units from the mean ELU richness for the state of Rhode Island. The statewide mean richness was 24 ELU types in the 457 m window. We consider any area that had more than 1 standard deviation (SD) more than the mean ELU richness to be highly variable. We reduced ELU richness into three discrete categories based on how many SD classes above the mean they were: class 1 is more than 1 SD above the mean variety (29–38 ELU types), class 2 is more than 2 SDs above mean variety (39–47 types of ELUs), and class 3 is >3 SD above mean variety (>47 kinds of ELU) (Table 3). For cartographic and aesthetic reasons, SD class areas were converted from raster format (15.24 m cell size) to vector polygons, their boundaries slightly smoothed to remove the jagged corners of pixels, and small or sliver polygons less than 0.4 ha removed by absorbing them into their surrounding polygons. We call these resulting three classes of ELU richness “planning classes” for use by non-scientific conservationists: Class 1 has a “good” variety of ELU types, class 2 has “better” ELU variety, and class 3 has the “best” richness of ELUs in Rhode Island.

**Table 3:** Standard deviation and resulting planning classes of the richness of ELUs within a 457 m (30 pixels) neighborhood.

Number of ELU types within 457 m (30 pixels)	Category	Area of land surface of RI (Sq Km, percent total state area)	Planning class
24	Mean variety for RI		
29–38	1 SD>mean	644.8 (23.1%)	Good
39–47	2 SD>mean	184.6 (6.6%)	Better
>47	3 SD>mean	28.4 (1%)	Best

### Testing the Relationship Between ELU Richness and Species Richness

We tested the null hypothesis that there was no relationship between ELU richness and plant species richness on the 24 Audubon Society of Rhode Island refuges. Refuges encompassed 16 different plant communities throughout the State of Rhode Island USA [17], [27]. Our measures of plant and community richness for each refuge were: total number of different ecological communities, total number of vascular plant species, and the total number of tree, shrub, and herbaceous plant species. For each refuge we measured the total number of different ELU types (ELU richness) that was found within the refuge boundaries.

Refuge size was significantly correlated with plant species richness (r = 0.63, p<0.0001), community richness (r = 0.53, p<0.001), and the number of different ELU types (r = 0.64, p<0.0001) on Audubon refuges. We removed this size bias by standardizing our measures of community, species, and ELU richness by refuge size (ha) thus providing estimates of ELU density (ELU types/ha), species density (number of species/ha), and community type density (number communities/ha) [28].

Because our measures of biodiversity and ELU richness were not normally distributed (Shapiro-Wilk Test), we used Spearman rank-order coefficient of correlation analysis to measure the degree of association between ELU richness and plant species richness. All statistical procedures were done using R software [33].

## Results

### Landscape-scale Patterns of ELUs

**Figure 2:**
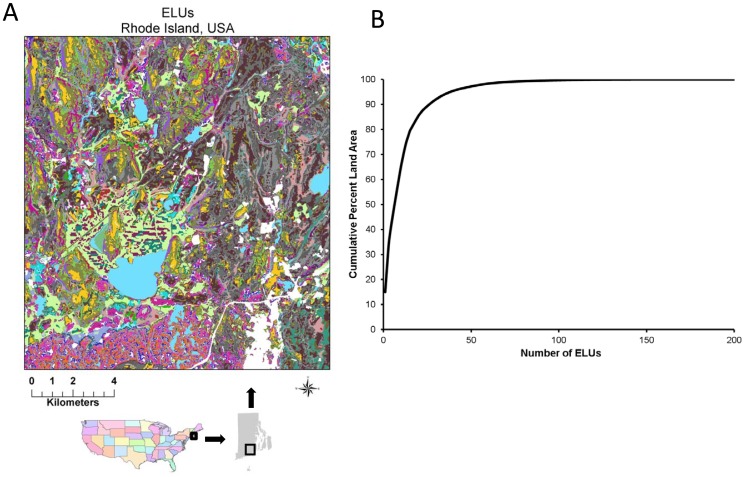
ELUs in Rhode Island. (A) Map showing ELUs for a Rhode Island landscape. (B) Cumulative distribution function for ELUs of Rhode Island.

There are 204 unique ELUs in Rhode Island, however, 85% of the land area of the state is covered by only 22 different ELUs (Table 2, Figure 2). The geography of ELUs clearly show the general landforms of the region such as the glacial moraine, large wetlands, upland forest in rolling hills, and glacial scouring of valleys and river channels.

**Figure 3:**
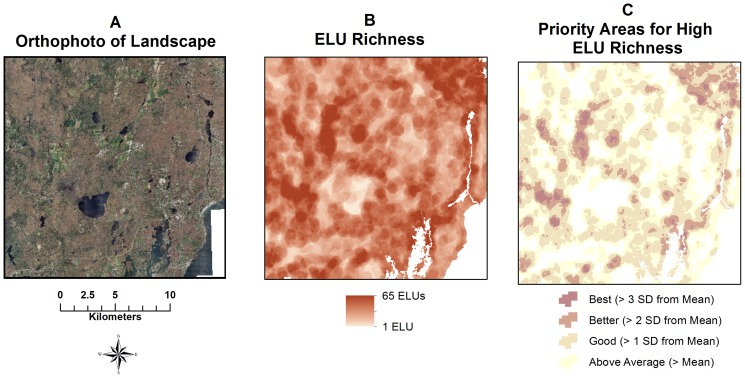
Spatial pattern of ELU richness. (A) Rhode Island landscape, 2011 digital orthophoto. Area mapped same as Figure 2. (B) Number of ELU types within a 30 pixel (457 m) radius. (C) ELU categories based on standard deviation units from the statewide mean variety ( = 24 ELU types in a 30 pixel radius). Basemap data from Environmental Systems Research Institute (Redlands, CA), and Rhode Island Geographic Information System database.

The spatial pattern of the richness of ELUs was not uniform and specific areas emerged as being highly variable (Figure 3). The simplification of the ELU richness map into three discrete planning categories based on SD units above the statewide mean resulted in clear patterns of ELU richness hotspots (Figure 3). River channels and wetlands frequently created high spatial diversity of ELUs because of the juxtaposition of well-drained and poorly-drained soils in a small area as well as large changes in topography and landform.

### Biodiversity and ELU Richness

**Figure 4:**
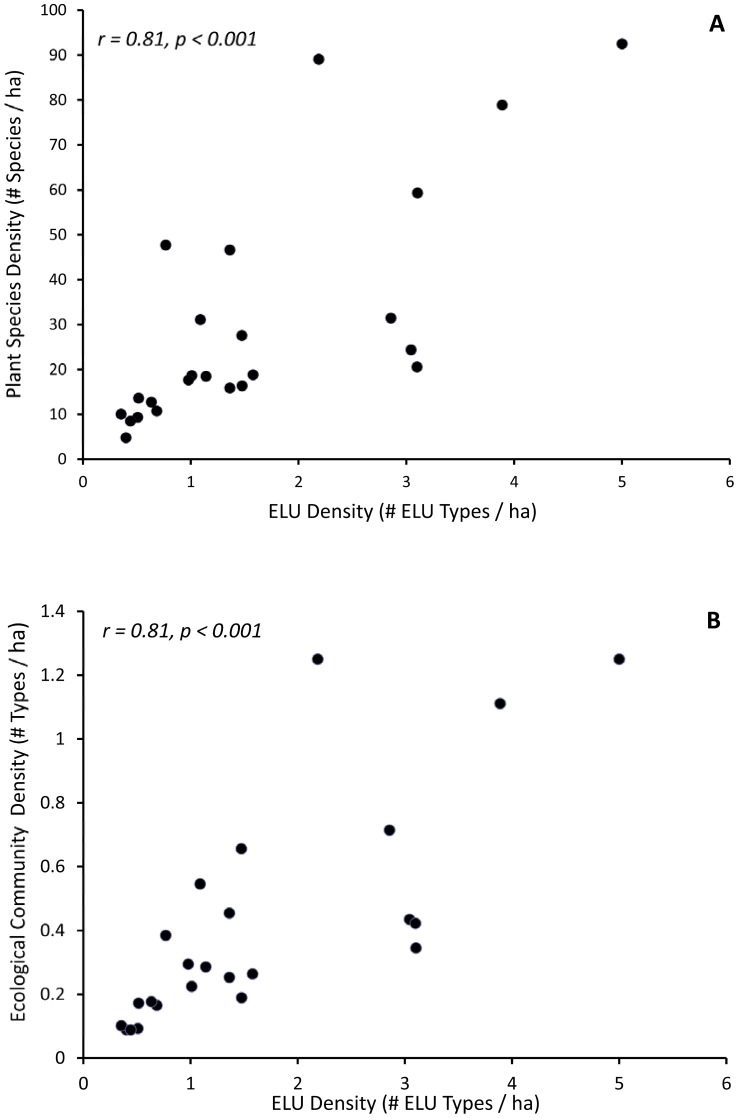
Total plant species and ecological community density versus ELU richness. (A) Total plant species density versus total number of different ELU types on ASRI refuges (standardized by refuge area in hectare). (B) Number of ecological communities on ASRI refuges versus ELU type density.

We hypothesize that landscapes with high ELU richness will support many different kinds of plant community types and result in high species richness of plants. This prediction is based on the assumption that unique ELUs may support unique plant communities; for example, wetland plant communities will occur on ELUs' containing poorly drained soils in valley bottoms whereas upland plant communities will occur in well-drained soils on hilltops and slopes. Our measures of plant species and community type richness were the species totals for Audubon refuges in Rhode Island standardized by refuge size to remove the bias of refuge area. All of the plant species density measures showed significant positive correlations with ELU density (herbaceous species, r = 0.80, p<0.001; shrubs r = 0.74, p<0.001; trees r = 0.60, p<0.005). There were significant positive correlations between ELU density and community type density (r = 0.81, p<0.001) and total plant species density (r = 0.81, p<0.001, Figure 4).

## Discussion

Ecological Land Units are a fundamental geophysical substrate of ecological communities and variation in geophysical settings can result in a diversity of plant associations in a region [13]–[15], [17]–[19], [34]. Our analysis indicates that spatial variation in ELUs is positively related to plant species richness and community diversity. Landscapes with high spatial variation in ELUs had greater plant biodiversity than landscapes with little variation in ELUs. Our study sites (Audubon Society of Rhode Island refuges) have been undisturbed for at least three decades and occur in a common climatic zone. Thus, factors that could have strong influence on biodiversity such as climate regime, land use history, pests, pathogens, and invasive species [35] were generally similar among sites. Our results support the hypothesis that landscapes that encompass many different geophysical settings (ELUs) show higher biodiversity than landscapes with homogeneous geophysical properties. This relationship spans multiple spatial scales over many biogeographic regions [13], [17]–[22], [34].

We modeled our ELU classification scheme after Anderson and Feree [13]. Instead of using bedrock geology as a basis for mapping ELUs we used USDA NRCS SSURGO data which are commonly available at very fine scales (typically 1∶12,000) for much of the United States. Bedrock data are coarsely mapped at small scale (1∶100,000) for Rhode Island and did not provide the spatial resolution of SSURGO soils for the state (1∶15,840). Furthermore, detailed digital bedrock data are not uniformly available across the United States as are the SSURGO data. Similarly, terrain data comparable to ours are readily available in the 1/3 and 1/9 arc second NED data available for much of the United States [25]. Thus, ELUs as we defined them can be mapped for most of the United States and other parts of the world where soils and terrain data are available. Our regions of high and low ELU richness are mapped at a resolution that is meaningful to local conservation organizations and integrate nicely with other geospatial data that are used to evaluate prospective properties to acquire. For example, ELU richness is one of many factors used by the Richmond Rural Preservation Land Trust (Town of Richmond, RI) to evaluate and rank prospective properties (Table 4).

**Table 4:** An example of land acquisition criteria used by a municipal land trust in Rhode Island showing how ELU richness is incorporated into a larger land protection context.

		Weighting Value (points)			
#	Criterion	0	1	2	3
1.	Size of parcel (acres)	<2 acre	2–25	25–50	over 50
2.	Groundwater/wellhead Protection	No impact	Non-community well	Recharge areas/community well	Aquifer
3.	Proximity to other protected lands	>½ mile	¼ to ½ mile	<¼ mile	Abutting or connecting such areas
4.	Proximity to water bodies	>½ mile	¼ to ½ mile	<¼ mile	Abutting or connecting such areas
5.	Natural habitat^1^	Degraded habitat	Average	Above average	Prime habitat
6.	Biodiversity value in future climates (ELU richness)^2^		Good	Better	Best
7.	Supports or is capable of supporting rare or endangered species^3^	No	May not fall w/in a Heritage Area, but exhibits qualities that could support r/e species	Falls w/in the Heritage Area of Special Concern	Documented proof of r/e species on property
8.	Farmland preservation	No	Inactive farm	Active farm <10 acres	Active farm >10 acres
9.	Potential to offset impact of development: # of housing units possible	0	1–10	11–20	over 20
10.	Historic value^4^	No	Yes		
11.	Protects rural character	No	Low	Medium	High
12.	Provides public passive recreational opportunities	No	Yes		
13.	Consistent with: a)Richmond comp. plan; b) Regional/Watershed Plan; c) Greenspace elements of State Guide Plan	0/3	1/3	2/3	3/3
14.	Price to Land Trust after other funding is considered	Market value	Below market value	Income generating	Donated

Different conditions for each criterion are assigned a weighted point value (0–3). The sum of the weights is totaled for a candidate property. Criteria provided by the Richmond (RI) Rural Preservation Land Trust.

1 As defined by the RI Natural History Survey.

2 As defined on ELU website.

3 As defined by the RI Natural Heritage Program.

4 Containing features defined by the RI Historical Society and/or Richmond Historical Society.

The practical implication of our results is areas of high ELU richness will likely support high biodiversity now and in the future when climate change results in significant modification in plant communities. Hunter et al. [12] demonstrated this relationship retrospectively and provided examples from the paleoecology literature where geophysically unique landscapes supported unique ecological communities over geological time. The Rhode Island climate in 2100 is expected to be similar to the current climatic regime of the southeastern United States [10]. Although there are many good modeling studies of the distribution of specific plant species under various climate change scenarios [1], there is considerable uncertainty in how species will respond to changes – adapt, disperse, go extinct – and what will be the resulting ecological communities [5]. This presents a conundrum for conservationists – where will future biodiversity hotspots be when plant (and animal) communities are significantly different due to a changing climate? Based on our results, we suggest that protecting areas of high ELU richness will be effective in protecting diverse ecological communities in the future.

Decisions to protect specific parcels of land are often made at local scales by conservation organizations that do not have the benefit of staff ecologists to advise them of the current scientific thinking on climate change and conservation [22], [35]. They must also balance other conservation goals of interest to constituents or donors, such as farmland or water resource protection. Therefore, we have endeavored to make our results meaningful, understandable, and accessible to conservation practitioners, especially land trusts and land conservancies. For example, the concept of standard deviation unit classes of ELUs relative to a statewide statistical mean is an abstract concept for a citizen conservationist on a land trust who does not have a strong background in ecology or statistics. However, “Good, Better, Best” categories (Table 3) are easy to understand and interpretable by anyone. We created a Rhode Island ELU web site (http://www.edc.uri.edu/elu) to explain the process of making ELUs, how they can be used, and how they correspond to patterns of biodiversity. The site contains downloadable GIS data for mapping ELUs, static maps of the geography of ELUs, and online mapping capability for local conservation practitioners.

ELUs are not the only driver of biological diversity [36]. Ecological pests, pathogens, land use history, dominance by invasive species, disturbance (or the lack of), and development can overwhelm the relationship between ELU diversity and species richness. Therefore, stewards of protected lands will be well-served, now and in the future, to be vigilant to invasive species, pests, and pathogens and be prepared to manage these sources of ecological disturbance on conservation properties and adjacent landscapes since they can reduce local levels of biodiversity regardless of the geophysical diversity of a site or region [37], [38]. At state and regional levels, ELU variety can serve as an additional criterion for setting site acquisition priorities. For municipalities and local land trusts, ELU richness is an objective, repeatable, transparent, and proactive criterion to help establish land planning and conservation priorities in the face of long-term climate change.

High species richness is only one of many possible biodiversity-based conservation goals. Others include protecting representative species and communities [39], [40], rare and endangered species [8], [15], [35], [41], landscape structures such as corridors or buffers [14], [39], [42], [43], and landscapes that provide unique or important ecosystem services [44]. ELUs can provide insight into some of these other conservation goals. For example, following the model of Zimmerman and Runkle [19] we were able to map under-represented ELUs in Rhode Island by comparing the prevalence of an ELU type on the landscape compared to how much of that ELU is included in the current portfolio of protected lands in the state. ELUs that are found infrequently on protected lands but occur with greater frequency on the overall landscape are geophysical settings that are underrepresented in our portfolio of protected lands and might be target for future conservation if representation is an important conservation goal.

Our maps of ELU richness have proven valuable in conservation planning over a broad range of scales. The ELU planning class maps are effective in identifying regions of high geophysical diversity statewide (2,500 sq km), as well as providing insight on ELU richness for specific properties at local scales (2.5 sq km). Furthermore, ELUs, as we have measured them, are broadly defined and can be mapped using the same constituent geophysical data over much larger regions than we have done in our study. If dispersal rates of plants and animals can keep pace with rates of climate change effects, corridors and islands of suitable ELU types for a given species or community type might represent the most parsimonious dispersal paths [5]. This is an aspect of ELU ecology and conservation planning that warrants further investigation.

## Supporting Information

Information S1 **Protocol to create Ecological Land Units (ELUs).** Procedures used to create ELUs. Methods taken from The Nature Conservancy metadata record for Northeastern United States ELU dataset.
(DOCX)
