# Supplementary material for: Conservation in the Context of Climate Change: Practical Guidelines for Land Protection at Local Scales
Source: PLoS One. 2013 Nov 20;8(11):e80874. doi: 10.1371/journal.pone.0080874 (PMC3835331; doi:10.1371/journal.pone.0080874)

Ecological Land Units (ELUs) as we have defined them is a composite of several layers of abiotic information: soil drainage class, soil texture, and landform. This supplemental document describes how ELUs are created. Much of it was taken from The Nature Conservancy metadata record for the Northeastern United States ELU dataset. A copy of the original metadata record can be obtained by contacting the senior author (K. Ruddock, kruddock@tnc.org).

Landform was derived from a 3 m digital elevation model (DEM) that was created from the 1997 digital terrain dataset available from RIGIS (http://www.edc.uri.edu/rigis/spfdata/elevation/5KDTM_terrain.zip). ArcGIS Spatial Analysis tools were used to create a slope raster from the DEM using the following slope classes: 0-2˚ (0.0-3.5%), 2-6˚ (3.5–10.5%), 6-24˚ (10.5–44.5%), 24-35˚ (44.5-70.0%), and >35˚ (>70.0%).

Landscape position index (LPI) is a unitless measure of the position of a point on the landscape surface in relation to its surrounding, is calculated for each elevation model point, as a distance-weighted mean of the elevation differences between that point and all other elevation model points within a radius of 304.8 m (1,000 feet, 20 pixels)

LPIo = [ ∑1,n (zi - zo) / di ] / n,

where zo = elevation of the focal point whose LPI is being calculated,

zi = elevation of point i of n model points within the specified search radius of the

focal point,

di = horizontal distance between the focal point and point i, and

n = the total number of model points within the specified search distance.

If the point being evaluated is in a valley, surrounding model points will be mostly higher than the focal point and the index will have a positive value. Negative values indicate that the focal point is close to a ridge top or summit, and values approaching zero indicate low relief or a mid-slope position.

The grid of continuous LPI values was subdivided into discrete classes of high, moderately high, moderately low, and low landscape position. These landscape positions and their corresponding LPI values are:

1. High landscape position (very convex): sharp ridges, summits, knobs. LPI < -0.076.
2. Moderately high landscape position: upper side slopes, rounded summits and ridges, low hills and convexities. LPI = -0.076 – 0.
3. Moderately low landscape position: lower sideslopes and toe slopes, gentle valleys and draws, broad flats. LPI = 0 – 0.068.
4. Low landscape position (very concave): steeply cut stream beds and coves, and flats at the foot of steep slopes. LPI > 0.068.

We assigned values 1-5 to the five slope classes, and 10, 20, 30, and 40 to the four LPI classes. We summed the grids to produce a matrix of values, and gave descriptive names to landforms that corresponded to matrix values as seen in Supplemental Figure 1. We collapsed all units in slope classes 4 and 5 into "steep" and "cliff" units, respectively.

Landform classes were reduced to 14 classes based on the matrix shown in Supplemental Figure S1.

Waterbodies from the RIGIS 1:5,000 hydrography dataset were incorporated into the landform layer as code = 51.

The ELU raster was created by summing the three rasters of landform, soil texture, and soil moisture as show in Table 1 of the paper. Every unique combination of these three geophysical features represents a unique ELU.

*Supplemental Figure S1: Formulation of landform models from land position and slope classes (from The Nature Conservancy metadata record for Northeastern United States ELU dataset).*


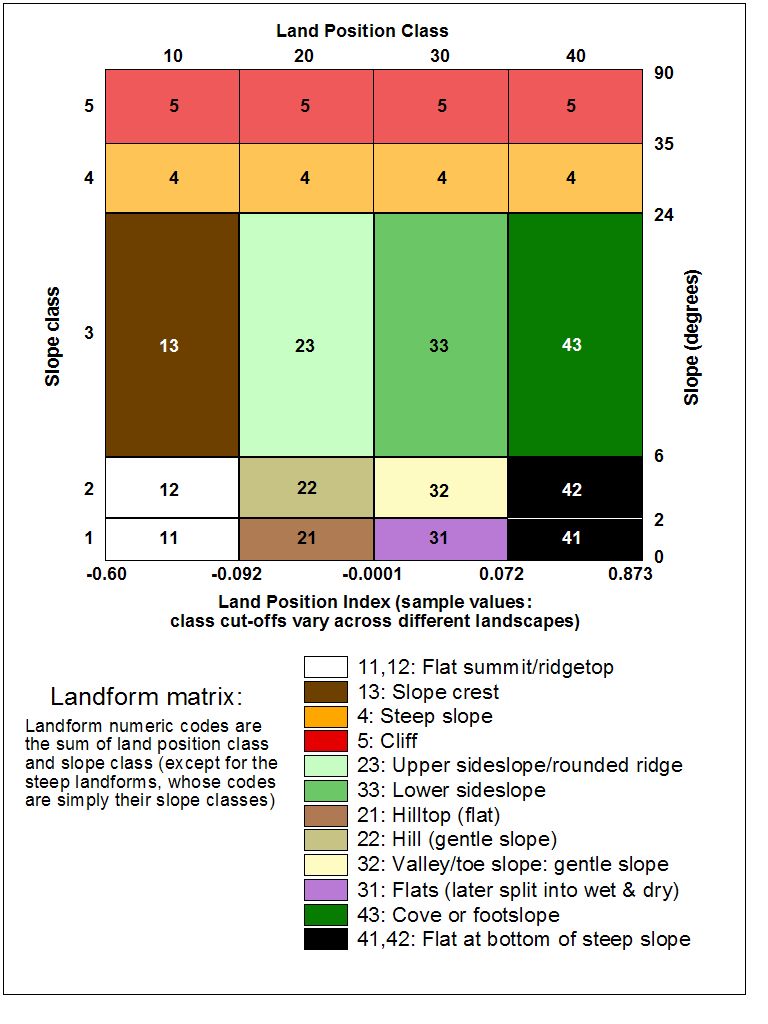

Supplement: Information S1 — Protocol to create Ecological Land Units (ELUs). Procedures used to create ELUs. Methods taken from The Nature Conservancy metadata record for Northeastern United States ELU dataset. (DOCX) [file pone.0080874.s001.docx]
